# Exosomes: small vesicles with big roles in hepatocellular carcinoma

**DOI:** 10.18632/oncotarget.10807

**Published:** 2016-07-24

**Authors:** Zhitong Wu, Qinghai Zeng, Ke Cao, Yifan Sun

**Affiliations:** ^1^ Department of Clinical Laboratory, Eighth Affiliated Hospital of Guangxi Medical University, Guigang City People's Hospital, Guigang, Guangxi, China; ^2^ Department of Dermatology, Third Xiangya Hospital, Central South University, Changsha, Hunan, China; ^3^ Department of Oncology, Third Xiangya Hospital, Central South University, Changsha, Hunan, China; ^4^ Department of Clinical Laboratory, Third Affiliated Hospital of Guangxi University of Chinese Medicine, Liuzhou, Guangxi, China

**Keywords:** hepatocellular carcinoma, exosomes, biomarker, function

## Abstract

Despite improvements in the diagnosis and treatment of hepatocellular carcinoma (HCC), the prognosis is still poor. Pioneering work has demonstrated a potential role for tumour cell-derived exosomes (TEXs) in HCC. TEXs can mediate immune responses, antigen presentation and intracellular communication by serving as vehicles for the transfer of proteins, viruses, lipids and RNA between cells. An improved understanding of the roles played by exosomes could lead to a powerful new strategy for preventing and treating HCC. In this review, we summarise current understanding on the topic. The literature points to two faces of TEXs in HCC: 1) They can promote invasion, metastasis, immune evasion and modulation and 2) they can act as diagnostic and prognostic biomarkers, and can be used in anti-cancer drug resistance and immunotherapy in the future.

## INTRODUCTION

Hepatocellular carcinoma (HCC) is a common cancer, with high morbidity and mortality, which places a heavy financial burden on sufferers, especially those in developing countries [1]. Despite developments in imaging, chemotherapy, interventional radiology, surgical techniques, and liver transplantation for HCC within the past two decades, the prognosis is still poor, and HCC remains the third most frequent cause of cancer-related deaths [2, 3]. At present, the best hope of improving the long-term survival of HCC patients is to detect the cancer at an early stage [4]. However, current noninvasive screening tools, such as serum α-fetoprotein and imaging (ultrasonography), are not highly sensitive, especially in the early diagnosis of HCC [5-7]. As with many other cancers, drug resistance, recurrence, and metastasis are the most important and difficult problems in the treatment of HCC [3].

Recently, tumor-derived exosomes (TEXs) have shown promise in the field of cancers [8]. The contents of TEXs, such as microRNA (miRNA) and oncoproteins, reflect the specific characteristics of their endosomal origin [9, 10]. TEXs are protected against degradation from enzymes (e.g., RNAse), as they are enclosed in a lipid bilayer [11]. Importantly, these enriched miRNAs and proteins in TEXs are selected, suggesting that the exosomal content may provide novel serological biomarkers for various types of cancer [12-14]. For example, research has suggested that glypican-1 could be used to detect early-stage pancreatic cancer [12] and that miR-21 and miR-93 could be used to detect early-stage HCC [13, 15]. In addition, TEXs can influence cancer progression and metastasis by the transfer of genetic materials between cells in the tumor microenvironment [16]. Thus, the evidence indicates that exosomes have various key roles in the development of cancer. An improved understanding of these roles may lead to the development of a powerful new strategy for preventing and treating HCC.

In this review, we summarize the roles and probable mechanisms of exosomes in HCC. In particular, considering the key role of miRNAs in cancer, we focus on exosomal miRNAs and their potential utility in HCC. The aim of the review is to illustrate the potential clinical applications of exosomes in the detection and treatment of HCC.

## GENERATION OF EXOSOMES IN THE LIVER

**Figure 1:**
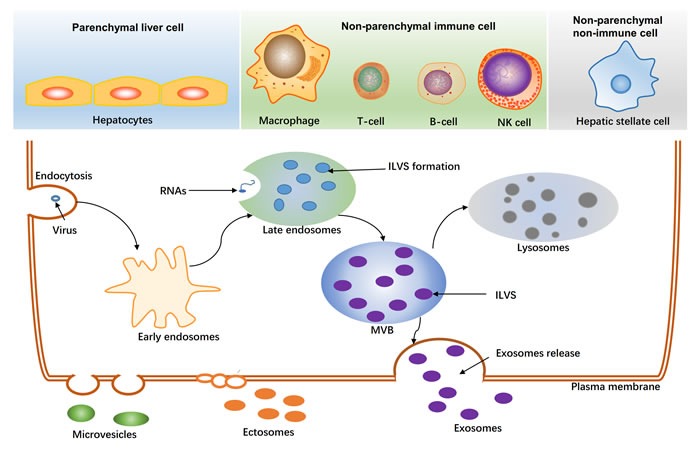
Generation of extracellular vesicles in liver The main three cell-types released extracellular vesicles in liver are shown at the top. The biogenesis of exosomes is shown at the bottom. Virus, proteins, RNAs are sorted to late endosomes, and further formatted intraluminal vesicles(ILVs). ILVs in the multivesicular body (MVB) released to the extracellular space by an exocytic step is regarded as exosomes (30-120nm). MVB can also be degraded as lysosomes. Ectosomes (100-350nm) and other microvesicles (100-1000nm) are budded from plasma membrane.

As shown in Figure 1, exosomes are formed by endocytosis, exocytosis, protein transport, and protein sorting. Virus and transmembrane proteins are endocytosed and transported into early endosomes. Following, early endosomes adevelop into late endosomes. In late endosomes, intraluminal vesicles (ILVs) form through inward budding of endosomal membranes and finally result in a large multivesicular body (MVB). ILVs in a MVB released into the extracellular space are referred to as “exosomes,” which typically have a size of 30-120 nm [17, 18]. Various types of cells, particularly tumor cells, can secrete exosomes into the extracellular matrix after multiple intracellular vesicles fuse with the cell membrane [17, 18]. Exosomes are found not only in serum, plasma, and urine but also in other body fluids, such as amniotic fluid, ascites, nasal lavage fluid, breast milk, saliva, and cell culture media [9, 15, 19-21].

With regard to the liver, three main cell types release exosomes (Figure 1): hepatocytes, nonparenchymal immune cells (e.g., Kupffer cells, natural killer cells, T cells, and B cells), and nonparenchymal liver cells (e.g., liver stellate cells). Exosomes derived from different cells in the liver have different functions, for example, exosomes derived from T cells and B cells are important mediators of inflammation [22], and exosomes derived from hepatic stellate cells may be involved in fibrosis processes [23].

## FUNCTIONS OF EXOSOMES IN HCC

### Exosomal miRNAs and HCC

Many studies have suggested that miRNAs participate in the development of HCC and that they may serve as potential prognostic or diagnostic markers for HCC [24-31]. The expression of the miRNAs miR-21, miR-221, and miR-222 is upregulated in HCC tissues [27-29], whereas the expression of miR-122a, miR-145, miR-199a, and miR-223 is downregulated, as compared to that of normal controls [27, 30, 32]. Recent studies suggest that exosomes may function as biological delivery vehicles for miRNAs [9, 33-35]. The expression of miRNAs in exosomes in HCC is shown in Table 1.

The miRNA of exosomes is different from that of their cells of origin. In miRNA expression profiling of Hep3B cells containing miRNA of exosomes derived from those cells, the expression levels of 25 miRNAs were significantly enriched in the exosomes, whereas those of 30 miRNAs were downregulated. Notably, 11 miRNAs were detected exclusively in the exosomes [9]. In another study of SMMC-7721 hepatocarcinoma cells, some miRNAs, such as miR-486-5p and miR-10b-5p, were highly abundant in exosomes but less abundant in cellular RNAs, and let-7d-5p, let-7b-5p, and let-7c-5p, which were highly abundant in cellular miRNAs, occurred only in very low numbers in exosomal RNAs [36]. These results point to selective enrichment of exosomes with specific miRNAs in HCC cells.

Exosomal miRNAs are transferable and functional in recipient cells. miR-122, a liver-specific miRNA that plays various roles in liver physiology and promotes the replication of the hepatitis C virus (HCV) [37], can be transferred *via* exosomes between two human hepatoma cell lines Huh7 and HepG2. When exosomal miR-122 released by Huh7 cells is transferred to HepG2 cells, it reduces the growth and proliferation of recipient cells [38]. Interestingly, exosomal miRNAs can also be transferred between different types of cells. Exosomes from adipose-derived mesenchymal stem cells (ADMSCs) can transfer miR-122 to HepG2 [35]. In addition, human and mouse liver cells, as well as primary human B cells, can exchange cellular endogenous miRNA, mediated by exosome shuffling [39]. miR-21, miR-192, and miR-221 in exosomes derived from colorectal cancer cell lines can be transferred to HepG2 cells and A549 lung cancer cells [33]. These findings indicate that miRNAs can shuttle between cells *via* exosomes, potentially promoting the invasion and metastasis of HCC.

Following the transfer of exosomal miRNAs to recipient cells, they regulate the target gene expression of recipient cells. miR-122-transfected ADMSCs can effectively package miR-122 into secreted exosomes and then deliver it to HCC cells, thereby altering the expression of miR-122 target genes, such as cyclin G1 and insulin-like growth factor receptor 1 in hepatoma cells [35]. In a previous study, the recurrence of HCC was higher in HCC liver transplantation patients with reduced expression of serum exosomal miR-718 [40]. The authors attributed the increased recurrence to miR-718 markedly suppressing the proliferation of Huh7 cells by upregulating the expression of the *HOXB8* gene.

**Table 1:** Specific expression of miRNAs in exosomes derived from HCC

**miRNAs**	**Source of exosomes**	**Source of compared**	**Function**	**Ref**
Upregulated miRNAs				
miR-584	Hep3B cells	Hep3B donor cells	Target TAK1, enhance transformed cell growth in recipient cells	[9]
miR-517c				
miR-378				
miR-520f				
miR-142-5p				
miR-451				
miR-518d				
miR-215				
miR-376a*				
miR-133b				
miR-367				
miR-21	Serum	CHB and healthy volunteers.	Correlated with cirrhosis and tumor stage	[15]
miR-486-5p	SMMC-7721 cells	HCC cells	Promote Proliferation and Metastasis	[36]
miR-10b-5p				
miR-18a,	Serum	LC and CHB patients	Novel serological biomarkers for HCC	[13]
miR-221				
miR-222				
miR-224				
Downregulated miRNAs				
let-7d-5p	SMMC-7721 cells	HCC cells	Promote Proliferation and Metastasis	[36]
let-7b-5p				
let-7c-5p				
miR-718	Serum	HOXB8	Target HOXB8, suppresses cell proliferation	[40]
miR-101	Serum	LC and CHB patients	Novel serological biomarkers for HCC	[13]
miR-106b	Serum	CHB patients	Novel serological biomarkers for HCC	[13]
miR-122				
miR-195				

CHB, Chronic Hepatitis B; LC, Live Cirrhosis;

### Exosomal proteomes and HCC

The content of exosomal proteins of hepatic cancer cell lines differs greatly, depending on their metastatic potential (high *vs*. low). In a quantitative analysis, specific proteins enriched in hepatocyte-derived exosomes included caveolae (caveolin 1), early endosomes, endoplasmic reticulum proteins, peroxisomes and mitochondria [41, 42]. Using mass spectrometry analysis, 213 unique proteins were detected in HCC-derived exosomes [43]. In the same study, a proteomic analysis revealed that the MET protein, caveolins, and S100 family members were significantly enriched in exosomes secreted by highly malignant HCC cells. Another study confirmed that the abundance of exosomal S100 family members, S100-A8 and S100-A9, significantly increased in response to HBV transfection in Huh-7 cells [44]. However, as shown by exosomal proteome profiling, the expression of these proteins does not seem to be specific to HCC. For example, the S100 protein is found in exosomes secreted from breast cancer cells [45], caveolin 1 is found in glioma [46], and MET is found in colorectal cancer cells [10]. MET signaling, caveolins and S100 family members are known to have pivotal roles in tumor growth, angiogenesis, and metastasis [47-49]. Thus, these exosomal proteomes could be common targets for the treatment of various types of cancer.

RNA binding proteins (RBPs) act as miRNA carriers in cells [50]. Intriguingly, some RBPs, such as high-density lipoproteins and argonaute-2 (Ago2), are present in exosomes [51, 52].The involvement of lipid-mediated RNA loading in exosomes has been demonstrated [53]. ELAVL1 is a member of the ELAVL family of RBPs. It directly interacts with long noncoding RNA (lncRNA), a target of miRNA, in HCC cells [54]. Ahadi et al. identified ELAVL1 motifs on exosomal lncRNAs, suggesting that ELAVL1 may also stabilize the transcript [55]. In addition, Schilders et al. [56] demonstrated that M-phase phosphoprotein 6 is an exosome-associated RBP for the recruitment of the exosome to pre-rRNA. Thus, the literature suggests that miRNA loading of exosomes may be based on the interaction of the RBP-RNA complex. Research has also shown that replication of the HCV was associated with the Ago2-HSP90-miR-122 complex in exosomes isolated from HCV patients or HCV-infected Huh7.5 cells [51]. Thus, based on the current literature, RBPs not only mediate the loading of miRNAs into exosomes, they also play critical roles in stabilizing these miRNAs in exosomes.

### Exosome-mediated invasion and metastasis

Tumor metastasis is a multistep process, which includes invasion, intravasation, and colonization of distal sites *via* the circulatory system [57]. The tumor microenvironment allows for tight integration of these processes [58]. Cancer-derived exosomes provide a means of cell-cell communication between cancer cells and may possibly recruit and reprogram the constituents of the tumor microenvironment [57]. The contents of exosomes, such as noncoding RNAs and proteins, may then influence cancer progression and metastasis [59].

Cellular TGF-beta-activated kinase 1 (TAK1) responds to environmental changes and intercellular modulation and is thought to contribute to the formation and growth of HCC [60]. In Hep3B cells, miRNAs are released into exosomes *via* a ceramide-dependent pathway, transferred to recipient cells, and then modulate the constitutive expression of TAK1 and downstream signaling associated with TAK1 in recipient cells [9]. Following the incubation of Hep3B and HepG2 with Hep3B-derived exosomes, the number of colonies of Hep3B and HepG2 cells in soft agar increasedFollowing ia, as well as the activity of caspase-3/7, indicating that exosomes have a positive effect on transformed cell growth and death *in vitro* [9]. CD90+ cancer stem cells are associated with metastasis and early recurrence in HCC [61, 62]. A previous study showed that CD90+ Huh7 cells derived from exosomes influenced the tumor microenvironment by promoting angiogenesis [63]. In that study, when endothelial cells were incubated with exosomes released by CD90+ Huh7 cells, the mRNA levels of the proangiogenic factor vascular endothelial growth factor (VEGF) and its receptor VEGF-R1 increased significantly in endothelial cells, in addition to adhesion between the CD90+ cells and endothelial cells. However, the aforementioned effects were not observed in exosomes released by Huh7 parental cells. Furthermore, H19, an lncRNA, was up-regulated (10-fold) in the exosomes derived from the CD90+ Huh7 cells compared with those derived from Huh7, suggesting that H19 lncRNA might stimulate angiogenesis and favor cell-cell interactions [63].

In common with noncoding RNAs, exosomal proteomes can promote the migration of cancer cells. Vasorin, a serum biomarker of hepatocarcinoma that is highly expressed in sera and tissues samples of hepatocarcinoma [64], is released by HepG2 cells in exosomes. Upon delivery of vasorin to human umbilical vein endothelial cells *via* exosomes, vasorin accelerates the migration of these cells [65]. In an immortalized hepatocyte line, the MET protein, caveolins, and S100 family members in HCC-derived exosomes triggered PI3K/AKT and MAPK signaling pathways and increased the secretion of matrix metalloproteinase-2 (MMP-2) and MMP-9, thereby facilitating the invasion activity of cancer cells [43].

### Exosome-mediated immune evasion

Viral miRNA or proteins can also be packaged into exosomes [66]. Epstein-Barr virus miRNAs can be delivered to uninfected cells *via* exosomes [67]. The HIV accessory protein negative factor stimulates its own export by releasing exosomes [68]. Hepatitis virus infection is a major cause of HCC. Upon release from host cells, the hepatitis A virus cloaks itself in exosomes, thereby protecting the virion from antibody-mediated neutralization [69]. Similarly, the release of HCV from infected cells is linked to the exosomal pathway [70], and exosomes from HCV-infected cells are capable of transmitting infection to naive human hepatoma Huh7.5.1 cells [71]. Interestingly, levels of extracellular infectious HCV are significantly lower in exosomes released from autophagy knockdown cells [72]. The aforementioned sheds light on the potential role of the exosomal pathway as an immune evasion mechanism, enabling the hepatitis virus to replicate efficiently in the liver. Studies of HBV virion morphogenesis and secretion in HBV-infected hepatocytes demonstrated that multivesicular bodies and related cellular proteins were significantly involved in this process. Although HBV infection is known to alter the contents of exosomes [73], exosome-mediated immune mechanisms in HBV infection and HBV-related HCC remain unclear. Further research is needed on exosomal HBV miRNA and miRNA transport in HBV infection.

## APPLICATIONS OF EXOSOMES IN HCC

### Exosomes for diagnosis

Exosome-associated miRNAs are more stable than nonexosome-associated miRNAs [74]. In addition, the expression of exosomal miRNAs is different to that of serum circulating miRNAs [75] and protein, as well as that of protein miRNAs [43]. Given the differences in the expression of these miRNAs, the exosomal content of cells can be used to aid the diagnosis of lung cancer [76], pancreatic cancer [12], and malignant melanoma [14]. As the miRNA and protein content of exosomes produced by HCC cells are completely different to those of normal cells [9, 36], with some miRNAs found only in HCC-derived exosomes [9], HCC-derived exosomes may serve as a novel diagnostic marker for HCC. Several exosomal miRNAs, such as miR-21, miR-18a, miR-221, miR-222, and miR-224, are candidate biomarkers [13, 15]. However, the diagnostic efficiency of exosomal miRNAs for HCC has not been determined. Furthermore, whether exosomal miRNA is a better biomarker than serum α-fetoprotein remains an intriguing topic for future investigations.

### Exosomes for drug resistance

Drug resistance is a major obstacle in the treatment of HCC, and novel strategies need to be developed to combat drug toxicity and sensitivity in chemotherapy. In this regard, exosomes have attracted much interest. In one study, cisplatin enhanced cytotoxic T lymphocyte-specific cytotoxicity elicited by exosomes when compared that of controls, and significantly prolonged the survival time of mice with HCC [77]. Another study proposed that miR-122 could modulate the sensitivity of HCC cells to chemotherapeutic drugs by downregulating multidrug resistance-related genes, antiapoptotic genes, and the cell cycle-related gene cyclin B1 [24]. More recently, ADMSC-derived exosomes were shown to deliver miR-122 to HCC cells [35]. *In vitro*, the percentage of the G0/G1 population of HepG2 and Huh7 cells increased significantly when incubated with chemotherapeutic agents and/or ADMSC-derived exosomes (122-Exo) [35]. In the same study, Huh7 cells treated with 122-Exo showed similar G0/G1 arrest trends, and these effects were confirmed *in vivo*. These findings indicate that 122-Exo can enhance cell apoptosis and cell cycle arrest and increase the chemosensitivity of HCC [35]. Based on the literature, exosomes seems to show major promise in combating drug resistance and sensitivity in chemotherapy for HCC.

### Exosomes as therapeutic tools

The function of exosomes as vehicles for the delivery of proteins and antigen chaperones illustrate their potential in immunotherapy of HCC [78]. For example, human macrophages can transfer miR-142 and miR-223 to HCC cells, decrease the expression of reporter proteins, and endogenously express stathmin-1 and the insulin-like growth factor-1 receptor, further inhibiting HCC cell proliferation or tumor growth [79]. Bone marrow stromal cells pulsed with homologous tumor-derived exosomes can increase the inhibition of homologous HCC H(22) cells, resulting in the arrest of H(22) cells in the G(0)/G(1) phase [80]. Exosomal proteins are actively released after stimulation. NY-ESO-1 is a tumor-specific antigen, with strong antigen immunogenicity [81]. The expression of the NY-ESO-1 protein in exosomes derived from HCC cell lines, as well as the expression of human leukocyte antigen, a tumor-specific immune-stimulating molecule, increases significantly after 5-Aza-2′-deoxycytidine treatment, indicating that exosomes secreted from hepatoma cells are able to stimulate an antitumor-specific immune response [82]. Notably, anticancer drugs also cause the release of exosomes from human HCC cells [83]. *In vitro*, after exposure to HCC cells resistant or sensitive to anticancer drugs, exosomes with superior immunogenicity were actively released by resistant anticancer drug-treated HepG2 cells, eliciting effective heat shock protein-specific natural killer cell antitumor responses [83]. In a rat model, exosomal ADMSCs facilitated HCC suppression by promoting NK T-cell anti-tumor responses [84]. These findings suggest that a vaccine that targeted exosomes could be efficient orin HCC immunotherapy.

## FUTURE PERSPECTIVES

**Figure 2:**
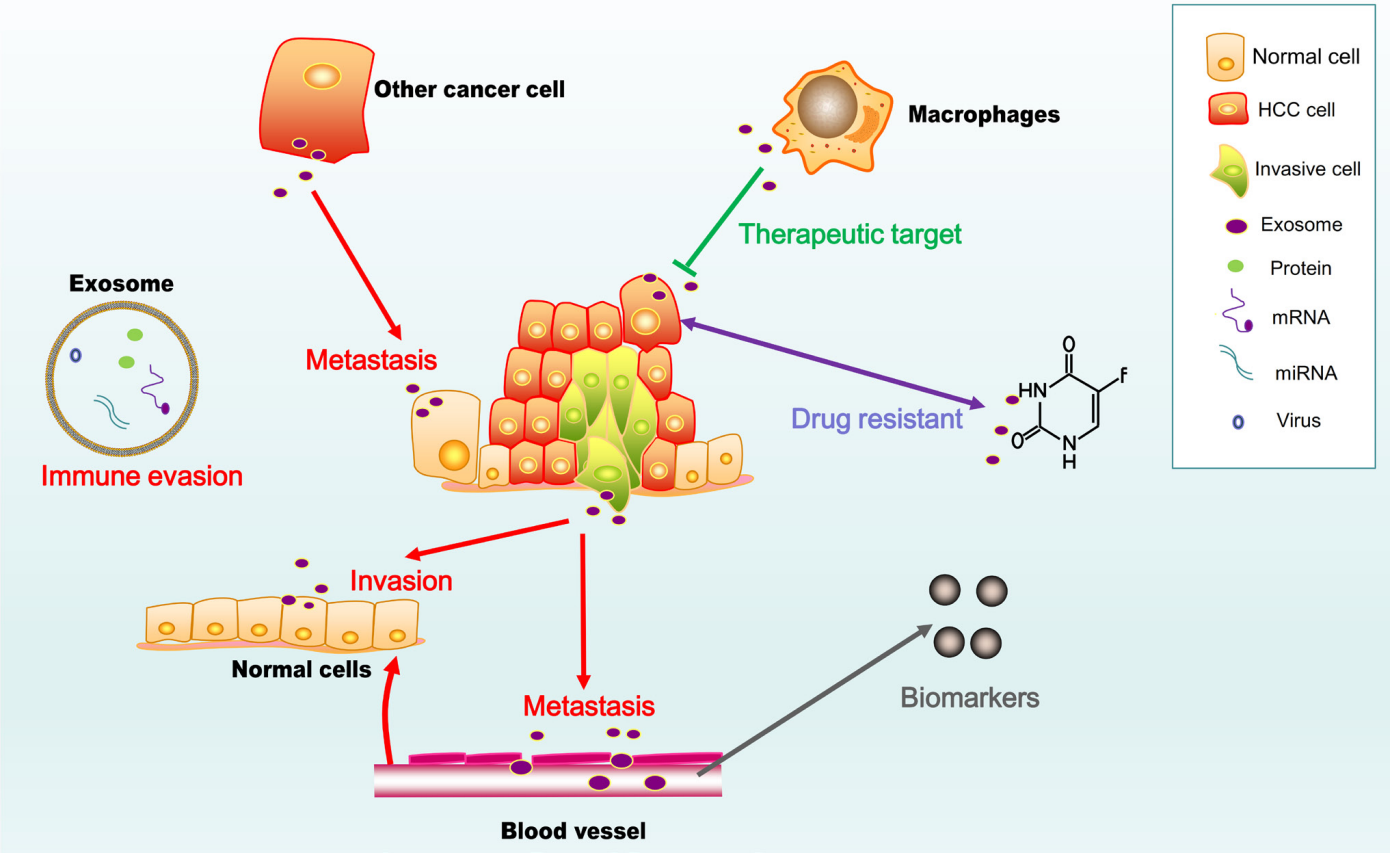
Two faces of exosomes in HCC The figure shows the pro-cancer activity of exosomes (marked in red), including their roles in invasion, metastasis and immune evasion, as well as their potential use as diagnostic and prognostic biomarkers and immunotherapy for HCC.

Considerable advances have been made in understanding the functions of exosomes in HCC (Figure 2). These advances facilitate the development of a powerful new strategy to prevent and treat HCC. However, to exploit the potential of HCC-derived exosomes, more studies are needed of some specific areas, as described below:

First, there are no current technical standards established at present for the purification and isolation of exosomes. For example, ectosomes, which are very similar to exosomes, are oproduced by all cells (Figure 1). Thus, distinguishing purified and isolated exosomes and ectosomes is problematic [18]. In addition, the heterogeneity of exosomes (i.e., different density of exosomes and different expression of exosomal proteomic markers) needs to be considered as this can affect the purification of exosomespurified [85]. Therefore, more discriminant techniques are needed for the purification and isolation of exosomes to obtain more homogeneous exosomal preparations.

Second, cancer-associated fibroblasts (CAFs) promote tumorigenesis, progression, invasion, and chemoresistance in the tumor microenvironment of cancers [86]. In our previous work, we showed that CAFs, as well as energy metabolism reprogramming and oxygen stress, played important roles in abnormal proliferation [87]. The progress of HCC is always associated with CAFs [88, 89], as most cases of HCC are the result of liver cirrhosis [90]. As HCC-derived exosomes are known to play important roles in the tumor microenvironment, the next challenge is to better understand the role of HCC-derived exosomes in CAFs, energy metabolism reprogramming, and oxygen stress.

Third, the epithelial-mesenchymal transition (EMT) mediates HCC invasion and metastasis [91], and miRNA inhibits the expression of proteins involved in the EMT and promotes the EMT in cancer cells [92, 93]. The role of exosomes in the promotion of the EMT was delineated recently [94]. Hence, new studies could focus on the possible role of enriched and specific miRNAs in HCC-derived exosomes in the EMT.

Fourth, in addition to miRNAs, lncRNAs mediate the progress of HCC [95]. Recent work showed that circular RNAs functioned as miRNA sponges [96]. No studies have investigated the functions of lncRNAs and circular RNAs in HCC-derived exosomes. Considering the important functions of miRNAs in exosomes from HCC cells, further studies should investigate the potential roles of lncRNAs and circRNAs in exosomes in HCC.

Finally, treatment based on exosomes may be a double-edged sword, given the pro- and anticancer-inducing activity of these extracellular vehicles. Therefore, numerous large studies should evaluate the clinical efficacy and safety of exosomes for use in the development of a vaccine for HCC immunotherapy.
